# Gastric-Type Cervical Adenocarcinoma: Clinicopathologic Features, Molecular Landscape, and Therapeutic Challenges

**DOI:** 10.3390/jpm16020072

**Published:** 2026-01-31

**Authors:** Hiroshi Yoshida, Daiki Higuchi, Waku Takigawa, Nao Kikkawa, Taro Yamanaka, Ayaka Nagao, Mayumi Kobayashi-Kato, Masaya Uno, Mitsuya Ishikawa, Kouya Shiraishi

**Affiliations:** 1 Department of Diagnostic Pathology, National Cancer Center Hospital, Tokyo 104-0045, Japan; 2 Division of Genome Biology, National Cancer Center Research Institute, Tokyo 104-0045, Japantayaman2@ncc.go.jp (T.Y.);; 3 Department of Gynecology, National Cancer Center Hospital, Tokyo 104-0045, Japan; 4 Department of Diagnostic Radiology, National Cancer Center Hospital, Tokyo 104-0045, Japan; 5 Department of Medical Oncology, National Cancer Center Hospital, Tokyo 104-0045, Japan; 6 Department of Radiation Oncology, National Cancer Center Hospital, Tokyo 104-0045, Japan; ayakatak@ncc.go.jp

**Keywords:** cervical cancer, gastric-type adenocarcinoma, clinicopathologic features, histology, immunohistochemistry, genetic alterations, therapeutic targets

## Abstract

Endocervical adenocarcinoma is now classified within an etiologic framework based on the presence or absence of high-risk human papillomavirus (HPV) infection. Gastric-type endocervical adenocarcinoma (GAS) is the prototypical HPV-independent subtype, accounting for up to 25% of endocervical adenocarcinomas and showing a particularly high frequency in East Asia. GAS is typically diagnosed at a more advanced stage than usual-type HPV-associated endocervical adenocarcinoma (UEA); exhibits deep stromal and parametrial invasion, lymphovascular space invasion, and a strong propensity for ovarian and peritoneal metastasis; and is associated with markedly worse survival, even in stage I disease. Radiological evaluation is challenging because of diffuse infiltrative growth, prominent mucin production, and frequent underestimation of extra-cervical spread. Histologically, GAS shows gastric-type (pyloric) differentiation, ranging from minimal deviation adenocarcinoma to poorly differentiated forms, and often overlaps with precursor lesions such as atypical lobular endocervical glandular hyperplasia and gastric-type adenocarcinoma in situ. Immunophenotypically, GAS is typically p16-negative, ER/PR-negative, and frequently exhibits mutant-type p53 and expression of gastric markers including MUC6, HIK1083, and claudin 18.2. Recent next-generation sequencing and multi-omics studies have revealed recurrent alterations in *TP53*, *CDKN2A*, *STK11*, *KRAS*, *ARID1A*, *KMT2D*, and homologous recombination-related genes, together with the activation of PI3K/AKT, WNT/β-catenin, TGF-β, and EMT pathways and characteristic metabolic reprogramming. GAS is highly resistant to conventional chemotherapy and radiotherapy, and its current management follows guidelines for squamous and usual-type adenocarcinoma. Emerging data support precision-medicine approaches targeting HER2/HER3, PD-1/PD-L1, and claudin 18.2, and suggest a role for PARP inhibition and other genotype-directed therapies in selected subsets. Given its aggressive biology and rising relative incidence in the HPV-vaccination era, GAS represents a critical unmet need in gynecologic oncology. Future progress hinges on developing reliable diagnostic biomarkers, refining imaging protocols, and validating targeted therapies through international clinical trials.

## 1. Introduction

The classification of endocervical adenocarcinoma (EAC) has recently shifted toward an etiologic framework based on the presence or absence of human papillomavirus (HPV) infection [1,2]. The latest WHO classification (5th edition, 2020) and the International Endocervical Adenocarcinoma Criteria and Classification (IECC) (2018) categorize endocervical adenocarcinomas into two major groups: HPV-associated adenocarcinoma (HPVA) and HPV-independent adenocarcinoma (HPVI) [1,3]. HPVA comprises the majority of endocervical adenocarcinomas (generally more than 80%) [1,2], arises from infection with high-risk HPV, and includes several major subtypes. The usual-type endocervical adenocarcinoma (UEA) represents the most common subtype within HPVA [2,3]. Additional subtypes include mucinous adenocarcinomas (such as intestinal and signet-ring cell types) and invasive stratified mucin-producing carcinoma (iSMILE) [2,3].

In contrast, HPVI encompasses adenocarcinomas unrelated to high-risk HPV infection [1,3] and accounts for approximately 10–25% of endocervical adenocarcinomas [2]. Gastric-type endocervical adenocarcinoma (GAS) is the most common HPVI subtype [1,2], and other HPVI subtypes include clear cell carcinoma and mesonephric carcinoma [1,2].

This review focuses on the current clinicopathologic understanding of GAS. In the WHO classification, GAS is defined as “adenocarcinoma, HPV-independent, gastric type, is an invasive adenocarcinoma showing gastric (pyloric) differentiation, unrelated to HPV infection” [2]. GAS is characterized by morphologic features resembling gastric or pancreatobiliary epithelium (gastric-type differentiation) [4,5,6], and encompasses a broad morphologic spectrum [7]. Historically known as adenoma malignum, minimal deviation adenocarcinoma (MDA) is now regarded as a well-differentiated subtype within the morphologic spectrum of GAS [4,5,6].

As GAS is unrelated to HPV infection, its relative incidence is expected to increase following the widespread implementation of HPV vaccination, which has led to reductions in squamous cell carcinoma and HPV-associated cancers [4,5,8,9,10]. Consequently, GAS is anticipated to become an increasingly important issue within the field of gynecologic oncology [4,5].

In this review, we summarize recent advances in the clinicopathologic features, molecular pathology, and therapeutic development of GAS. We aim to enhance awareness of this highly refractory malignancy and to provide a foundation that may stimulate new research efforts, including prevention strategies and novel therapeutic approaches.

## 2. Clinical and Imaging Features of GAS

### 2.1. Epidemiology and Incidence

According to the most recent global statistics, cervical cancer is estimated to affect approximately 660,000 women and to cause about 340,000 deaths annually worldwide [11], and it remains a significant health concern for women. Approximately 20–25% of cervical cancers are adenocarcinomas [1,4,12], and GAS accounts for 10–25% of these adenocarcinomas [2]. The incidence of GAS shows geographic variation; in Japan and other Asian countries, its proportion is relatively high, accounting for 20–25% of all ECA [13,14]. In one Japanese study, 328 cases of ECA were evaluated, and a total of 95 of the 328 tumors were classified as GAS [13]. In contrast, in Western countries, GAS is reported to account for approximately 10% of all ECA [1]. The reasons for these apparent racial and ethnic differences in the frequency of GAS remain to be elucidated.

### 2.2. Clinical Presentation and Prognosis

GAS is a major subtype of HPVI [2,4,14] and has been reported to show clinical behavior that is clearly distinct from that of UEA, with more aggressive features and poorer outcomes [4,13,15,16]. Patients with GAS tend to be slightly older at diagnosis than those with UEA [4,13,15,17,18]. In many studies, the median age of patients with GAS has been reported to be approximately 51 years (range, 27–83 years) [15,16]. In one comparative analysis, the mean age of patients with UEA was 47 years, whereas that of patients with GAS was 51 years [15]. However, a substantial proportion of patients with GAS are relatively young, and some cases are detected in the context of abnormal pregnancy courses [19]. The clinical manifestations of GAS are often nonspecific, making diagnosis particularly difficult in the early stages [17,20,21]. In general, patients present with symptoms similar to those of HPV-associated cervical carcinoma, including abnormal vaginal bleeding, pelvic pain, and abnormal Pap smear findings. In addition, it has been reported that up to 50–70% of patients complain of profuse watery vaginal discharge as their chief symptom [22,23].

Since current screening strategies are increasingly shifting toward primary HPV testing, there is a significant risk that GAS will be missed due to its HPV-independent nature. In this context, cervical cytology remains valuable; not necessarily for definitive diagnosis, but for detecting abnormalities such as ‘atypical glandular cells (AGC),’ which serve as a critical trigger for further investigation using histology and biomarkers [24,25,26]. Interestingly, a subset of GAS cases has been reported in association with Peutz–Jeghers syndrome (PJS) [25,27,28,29,30]. PJS is caused by germline mutations in the *STK11*/*LKB1* tumor suppressor gene [25]. An association between PJS and GAS has been described, often presenting with precursor lesions like lobular endocervical glandular hyperplasia (LEGH) [31,32,33]. In addition, rare cases of GAS have been reported in association with Li-Fraumeni syndrome [4]. Although routine blood tests show no specific abnormalities, an elevated serum CA 19–9 level has been observed relatively frequently, in approximately 50% of patients [15,18,21].

GAS tends to be diagnosed at an advanced stage and exhibits a distinctive pattern of metastasis that differs from that of UEA [4,13,15,16,34]. In contrast to UEA, which is detected predominantly at FIGO stage I [4,13,15,16], a large proportion of patients with GAS, 59% in one study [4] and 73.8% in another [15], are diagnosed at an advanced stage (FIGO stage II or higher) [4,13,15,16,35]. Compared with UEA, deep stromal invasion, lymphovascular space invasion (LVSI), and parametrial invasion are observed more frequently [13,15]. Lymph node metastasis is present at diagnosis in approximately 50% of patients [4,13,15], which is significantly higher than the 12% lymph node metastasis rate reported for HPVA [15]. Furthermore, unlike UEA, which more often remains confined to the pelvis [4], GAS shows an early tendency to metastasize to the peritoneum [4,13,15,16], omentum [4,13,15], adnexa (ovaries), and other extrauterine distant sites [4,7,13,15]. The reported frequency of ovarian involvement at diagnosis ranges from 5.3% [13] to 35% [4], which is higher than that in UEA [4,13,15]. Ovarian metastasis has been identified as an independent predictor of poor outcomes [16]. The rate of positive peritoneal washing cytology (PWC) at the time of surgery is also high in GAS, at 24.0% [18], suggesting early intraperitoneal dissemination. Positive PWC findings have been reported even in cases of gastric-type adenocarcinoma in situ (gAIS) [18]. In addition to the abdomen and omentum, metastatic spread to distant organs such as the liver, brain, bone, abdominal wall, and appendix has been documented [4,7], in clear contrast to the metastatic pattern typically seen in UEA [4,7]. These distinctive patterns of diffuse invasion and early metastasis reflect the aggressive biological phenotype of GAS, driven by molecular alterations such as *TP53* mutations and activation of epithelial–mesenchymal transition (EMT) pathways, which promote high cellular motility [36,37,38]. At present, GAS is staged according to the same FIGO system used for UEA and squamous cell carcinoma (SCC) [39], but in light of its unique biological characteristics, the appropriateness of this approach needs to be critically evaluated [4,16,18].

Multiple studies have consistently demonstrated that GAS is associated with poorer outcomes than UEA [4,13,14,15,16]. In one study, across all stages, the 5-year overall survival (OS) rate for GAS was 47.9%, compared with 82.5% for HPVA [15]. In another study, the 5-year disease-specific survival (DSS) rate for GAS was 42%, which was markedly worse than the 91% observed for UEA [4]. Even when analysis is restricted to stage I disease, GAS has been shown to have poorer outcomes than UEA [4,13,15,16]. The 5-year DSS rate for stage I GAS was 62%, significantly lower than the 96% reported for UEA [4]. For patients with stage II or higher GAS, median progression-free survival (PFS) and OS have been reported as 17 months and 33 months, respectively [16]. In a small series, the mean OS and mean disease-free survival (DFS) for GAS were 20.9 months and 12.2 months, respectively, both significantly shorter than those for UEA [20].

Although MDA is morphologically well differentiated, its clinical behavior remains a matter of ongoing debate. Historically, MDA was considered to be as aggressive as non-well-differentiated GAS, with studies showing no significant difference in stage distribution or survival between the two [4]. In contrast, recent reports suggest that MDA might be diagnosed at an earlier stage and associated with a more favorable prognosis compared to conventional GAS [40]. These conflicting data highlight the need for further studies to determine whether MDA represents a distinct prognostic subgroup.

In addition, GAS resistance to conventional treatment modalities is considered one of the principal factors underlying its poor outcomes [4,13,15,16,34]. Several studies have confirmed that GAS exhibits resistance to both chemotherapy and radiotherapy [34,35,41].

### 2.3. Imaging Findings of GAS

GAS has been reported to exhibit distinctive imaging features that differ from those of conventional cervical carcinomas, such as SCC and UEA [42,43,44,45]. However, the accuracy of preoperative imaging-based staging is limited [46], and there is a particular tendency for GAS staging to be underestimated compared with UEA [46].

**Figure 1:**
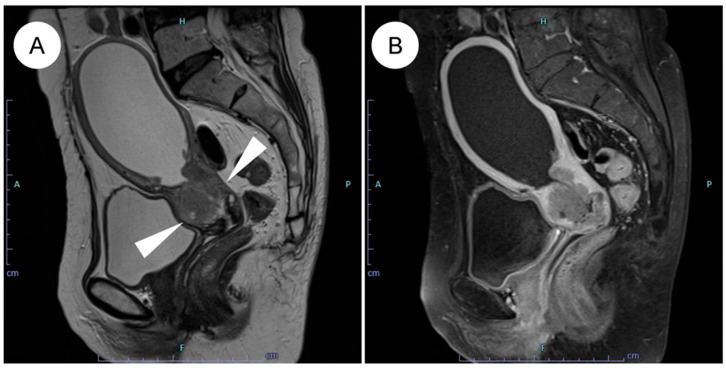
MRI features of HPV-independent gastric-type endocervical adenocarcinoma. (**A**) On sagittal T2-weighted imaging, an endophytic, mildly hyperintense mass is seen in the upper cervix (white arrowheads), showing diffuse infiltrative growth with small intratumoral cysts and associated hydrometra. (**B**) On post-contrast T1-weighted imaging, the tumor demonstrates mild enhancement. Note the mild enhancement compared to the typically more avid enhancement seen in usual-type adenocarcinoma.

Magnetic resonance imaging (MRI) is regarded as the optimal modality for assessing local invasion in cervical cancer [47], and several characteristic MRI findings have been described for GAS (Figure 1) [42,43,44,45]. (1) Tumor location: the tumor often involves the entire cervix, including the upper portion (13 of 18 cases) [42]. SCC and UEA tend to be localized predominantly to the lower cervix, including the squamocolumnar junction (SCJ), where HPV infection arises [42,43]. By contrast, because LEGH, a potential precursor lesion of GAS, is frequently located in the upper endocervical canal, GAS also tends to extend into the upper cervix [5,42,48]. (2) Growth pattern: a diffuse infiltration pattern is the predominant pattern of growth (17 of 18 cases) [42], whereas SCC and UEA generally show a mass-forming pattern [42]. This inwardly expanding, deeply infiltrative pattern reflects the highly aggressive nature of GAS and may contribute to underestimation of the true extent of disease on imaging [42,43,44,46]. (3) Associated cystic changes: microcysts (≦3 mm) or macrocysts (>3 mm) are frequently observed (14 of 18 cases) [42], reflecting the highly mucinous (mucin-producing) nature of GAS [42]. In some cases, GAS exhibits a “cosmos pattern,” similar to that seen in LEGH, characterized by clusters of microcysts centrally and surrounding macrocysts peripherally; in such situations, differentiation from LEGH based on MRI alone can be challenging [42,49,50]. (4) Solid tumor formation: although GAS was previously recognized mainly as a multilocular cystic mass, it is now appreciated that most cases appear predominantly as solid tumors on MRI [42,43,45,49]. (5) Intrauterine fluid collection: intrauterine fluid collection is frequently observed (13 of 18 cases) [42]. This finding is thought to result from inward tumor growth with diffuse stromal infiltration, which reduces cervical tissue compliance and narrows the endocervical canal, together with copious mucin production [42].

It has also been reported that the apparent diffusion coefficient (ADC) values of GAS are higher than those of SCC [42,43,44]. These elevated ADC values may reflect a T2 shine-through effect caused by mucin within cysts and dilated glands [42]. In addition, they may be related to the histologic finding that tumor cells are sparsely distributed within a fibrous stroma [42], which can reduce conspicuity on diffusion-weighted imaging (DWI) and potentially compromise the accuracy of tumor extent assessment [42,46].

Furthermore, limitations in diagnostic performance for staging have been highlighted [46]. Compared with UEA, GAS has been shown to be significantly understaged by preoperative imaging (integrated MRI/CT/PET-CT) [46]. On MRI, the sensitivity for detecting parametrial invasion is 0.49, for vaginal stromal invasion it is 0.54, and for adnexal involvement (including ovarian metastasis), it is only 0.15, indicating very low sensitivity [46]. Although FDG PET-CT is generally considered the most accurate modality for diagnosing lymph node metastasis [42,44,45,46], its performance in GAS is limited [46]. The sensitivity for lymph node metastasis (LNM) is low, with values of 0.48 for pelvic lymph node metastasis (PELNM) [46], and 0 for para-aortic lymph node metastasis (PALNM) [46]. In one study, 6 of 58 patients had PALNM, yet none of these metastases were detected by preoperative imaging [46]. The sensitivity for peritoneal dissemination was likewise low, at 0.25 [46].

These challenges in imaging-based diagnosis of GAS are largely attributable to its highly infiltrative growth pattern and prominent mucin production, and it has become evident that conventional imaging criteria do not permit staging with the same level of accuracy as in UEA or SCC. Therefore, in determining treatment strategies for GAS, it is essential to recognize the limitations of imaging and to develop diagnostic approaches that integrate molecular markers with characteristic imaging findings.

## 3. Pathological Characteristics of GAS

### 3.1. Histopathological Features

#### 3.1.1. Invasive Carcinoma

**Figure 2:**
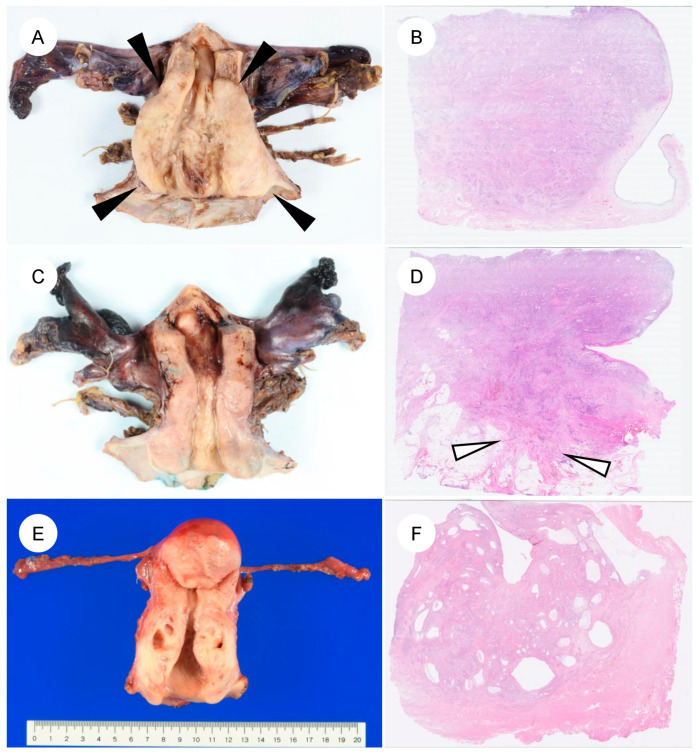
Gross and corresponding panoramic histologic findings of gastric-type endocervical adenocarcinoma. (**A**–**F**): Macroscopic appearance and corresponding panoramic views of gastric-type endocervical adenocarcinoma. (**A**) The cervix shows circumferential, diffuse mural thickening (black arrowheads) without any exophytic growth into the uterine cavity. (**B**) Panoramic view of the corresponding H&E-stained section demonstrates replacement of the markedly thickened cervical wall by an infiltrating carcinoma. (**C**) Another case showing circumferential cervical wall thickening, similar to the tumor in (**A**). (**D**) On the panoramic view, the tumor is seen extending into the parametrial soft tissue (white arrows). (**E**,**F**) A case of gastric-type adenocarcinoma detected during follow-up for lobular endocervical glandular hyperplasia. Multiple cysts are evident within the cervical wall.

The pathological features of GAS differ markedly from those of UEA [4,5,13,22,23]. A characteristic feature of GAS is its broad morphologic spectrum, ranging from extremely well-differentiated MDA to more cytologically atypical adenocarcinoma [4,5,6,7]. Macroscopically, GAS is often identified as a diffusely infiltrative lesion without a well-defined border, reflecting its highly invasive growth (Figure 2A–D) [4,13,45]. The tumor frequently involves the entire cervix, including the upper endocervical canal (high cervical canal) [4]. In some cases, the cervix is enlarged and indurated, producing a so-called “barrel-shaped cervix” [4,13,51]. On cut surface, the tumor often appears gray-white or gray-yellow and commonly contains cystic components (a multiloculated mass), which can impart a honeycomb-like appearance (Figure 2E,F). This morphology reflects the profuse mucin production characteristic of GAS [5,45,51]. The gross appearance of MDA can at times be challenging to recognize as neoplastic; the cervix may appear macroscopically normal or show only mild enlargement and induration, which contributes to the diagnostic difficulty [4,5,7,51].

**Figure 3:**
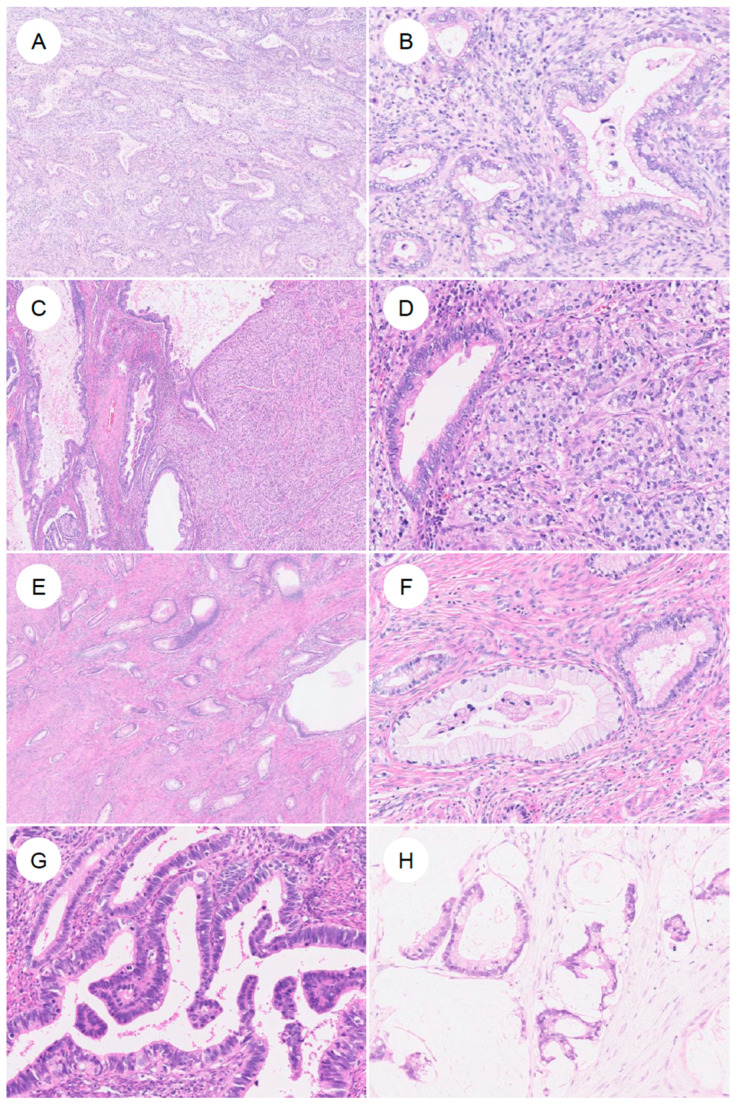
Histologic spectrum of gastric-type endocervical adenocarcinoma. (**A**) Typical gastric-type adenocarcinoma. At low magnification, irregular, angulated glands infiltrate the cervical stroma, accompanied by desmoplastic stromal reaction and inflammatory cells. (**B**) At higher magnification, the neoplastic glands are lined by adenocarcinoma cells with enlarged nuclei and pale to lightly eosinophilic mucinous cytoplasm, showing sharply defined cell borders. (**C**,**D**) In some tumors, in addition to well-formed glandular areas (left), a solid growth component (right) is also present. (**E**,**F**) A subset of tumors shows a highly differentiated morphology, composed of single, small tubular glands infiltrated with minimal or absent stromal reaction. The nuclear-to-cytoplasmic (N/C) ratio is low and cytologic atypia is mild; such tumors have historically been referred to as minimal deviation adenocarcinoma (formerly adenoma malignum). (**G**) More recently, it has been recognized that some gastric-type adenocarcinomas may contain components that closely resemble usual-type endocervical adenocarcinoma. (**H**) In rare cases, tumors exhibit a mucinous carcinoma-like pattern with abundant extracellular mucin.

Histologically, GAS is characterized by morphologic features resembling those of the stomach (pyloric glands) or pancreatobiliary tract, that is, gastric-type differentiation, and exhibits a spectrum of appearances ranging from well- to poorly differentiated forms (Figure 3) [7]. The tumor cells have abundant, voluminous cytoplasm, which is clear to lightly eosinophilic, and show distinct cell borders [7,13,14]. The cytoplasm is sometimes foamy or mucin-rich and appears clear [5,7]. The nuclei are usually basally located and, unlike the elongated, pseudostratified nuclei of UEA, are round to oval with vesicular nuclei and prominent nucleoli [7,14]. The spectrum of cytologic atypia is broad, ranging from lesions with minimal atypia, as seen in MDA (bland nuclear morphology), to those with moderate to marked atypia and poorly differentiated tumors showing signet-ring cell-like morphology [7]. GAS also exhibits marked intratumor heterogeneity, often encompassing multiple histologic patterns within the same tumor [7]. Preoperative biopsy specimens of GAS are often challenging to diagnose accurately, owing to factors such as high-grade differentiation and a low volume of identifiable tumor cells [35,52]. In a review on Western case series, GAS was suspected on preoperative biopsy in only 25–42% of cases [35]. In addition, because the tumor may be located high in the endocervical canal and extend into the uterine corpus, it can be misdiagnosed as endometrial carcinoma [53,54]. The neoplastic glands typically exhibit irregular shapes and sizes, with infiltrating glands often assuming a “claw-like” configuration [4,7,50]. Cribriform architecture and papillary proliferations protruding into the glandular lumen are also observed [4,7]. The stromal response around invasive glands is another characteristic feature; desmoplastic stromal reaction surrounding the infiltrating glands is frequently present [4]. However, in MDA in particular, this stromal reaction may be subtle and focal, or even virtually absent [4,6,7]. With respect to mitoses and apoptosis, UEA typically show readily identifiable apoptotic bodies and apical mitotic figures at scanning magnification [5,7]. In contrast, in GAS, these features are few, or when present, may not be readily recognized [5,7]. Nonetheless, in rare cases, GAS may exhibit glandular structures with elongated, pseudostratified nuclei resembling those of UEA, creating diagnostic difficulty [5,7]. MDA occupies the most highly differentiated extreme of the GAS spectrum and has historically been referred to as adenoma malignum [4]. Under the current WHO classification, MDA is viewed not as a distinct biological entity, but as part of a continuous morphologic spectrum with typical GAS. In a study that defined MDA as tumors in which more than 90% of the lesion shows a well-differentiated, low-grade morphology [4], MDA, compared with conventional GAS, exhibited only mild cytologic atypia [4], well-differentiated glands infiltrating deeply in a disorderly fashion, minimal stromal reaction, abundant apical mucin, and largely preserved nuclear polarity. While historically considered as aggressive as typical GAS, recent studies suggest that pure MDA might be associated with better clinical outcomes [40], although this remains a matter of ongoing debate. Consequently, strictly distinguishing MDA from GAS is often challenging, and they are currently categorized within the same disease entity.

In addition, a microcystic, elongated, and fragmented pattern has also been reported [7]. Diverse lines of differentiation further characterize GAS. Intestinal-type differentiation with goblet cells has been described [4], and, more rarely, squamous differentiation [55,56] and a primitive enterocyte phenotype [57] have been reported, resulting in a wide variety of histologic appearances. In both GAS and LEGH, tumor cells are known to contain neutral mucin in the cytoplasm and to show PAS-positive staining [58,59].

#### 3.1.2. Precursor and Preinvasive Lesions

**Figure 4:**
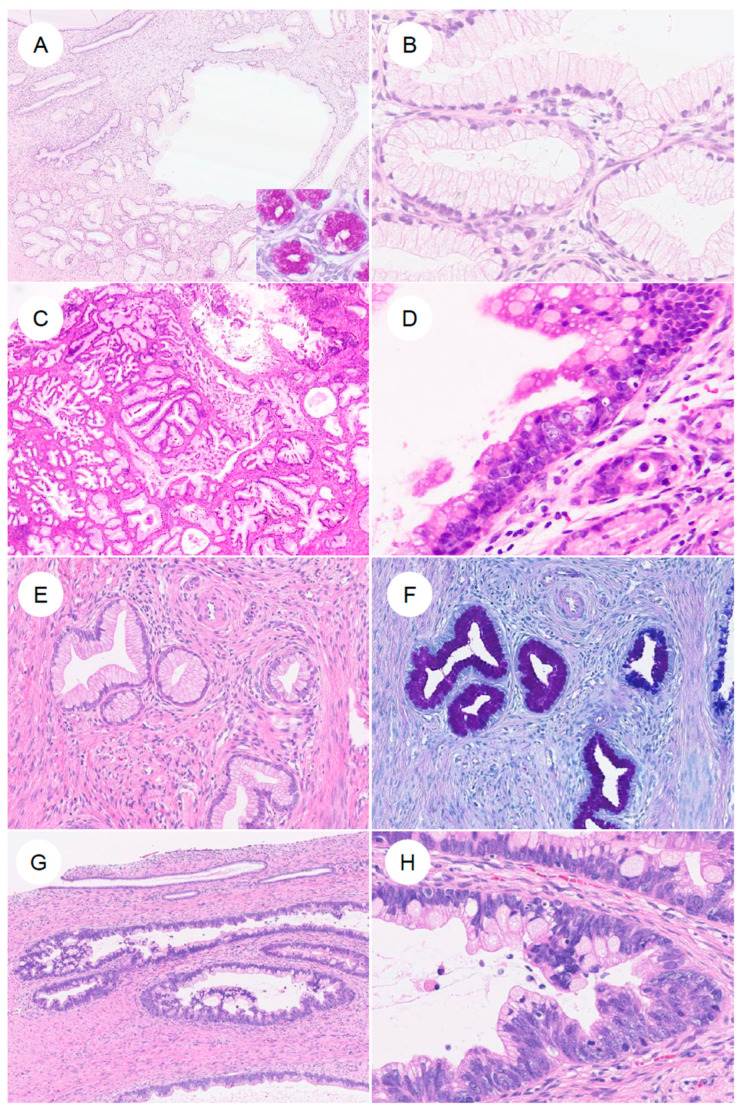
Precursor lesions and in situ neoplasia in gastric-type endocervical adenocarcinoma. (**A**,**B**) Lobular endocervical glandular hyperplasia (LEGH). At low magnification, large cystically dilated glands are present, surrounded by clusters of smaller glands arranged in a lobular configuration. (**B**) At higher magnification, the glandular epithelium shows abundant mucinous cytoplasm with small nuclei aligned in an orderly fashion along the basal aspect. (**C**,**D**) Atypical LEGH. At low power, the overall lobular architecture of LEGH is retained. At higher magnification, cytologic atypia is evident, including nuclear enlargement, irregular nuclear contours, loss of nuclear polarity, conspicuous nucleoli, coarse chromatin, mitotic figures, and apoptotic bodies. (**E**,**F**) Pyloric gland (gastric-type) metaplasia. Single, small tubular glands without a conspicuous lobular architecture as seen in LEGH, containing neutral mucin that stains red with AB–PAS. (**G**,**H**) Gastric-type adenocarcinoma in situ (gastric-type AIS). Large, angulated glands are lined by markedly atypical epithelium with nuclear enlargement, coarse chromatin, and prominent nucleoli.

Noninvasive lesions in the spectrum of GAS include LEGH and pyloric gland metaplasia, which are benign lesions, as well as atypical LEGH and gAIS arising from them (Figure 4) [7,60,61,62,63]. Although the diagnostic criteria for gAIS have not been fully established, the essential criteria in the 2020 WHO classification are described as “proliferation confined to normal endocervical glands; glandular epithelium with distinct cell borders and eosinophilic to pale mucinous cytoplasm; nuclear atypia and proliferation; intraglandular complexity allowed; negative/patchy p16 or negative HPV testing; negative ER and PR” [2], and confirmation of positivity for HIK1083, CK7, and MUC6 (MUC6 is nonspecific) is considered desirable [2].

**Figure 5:**
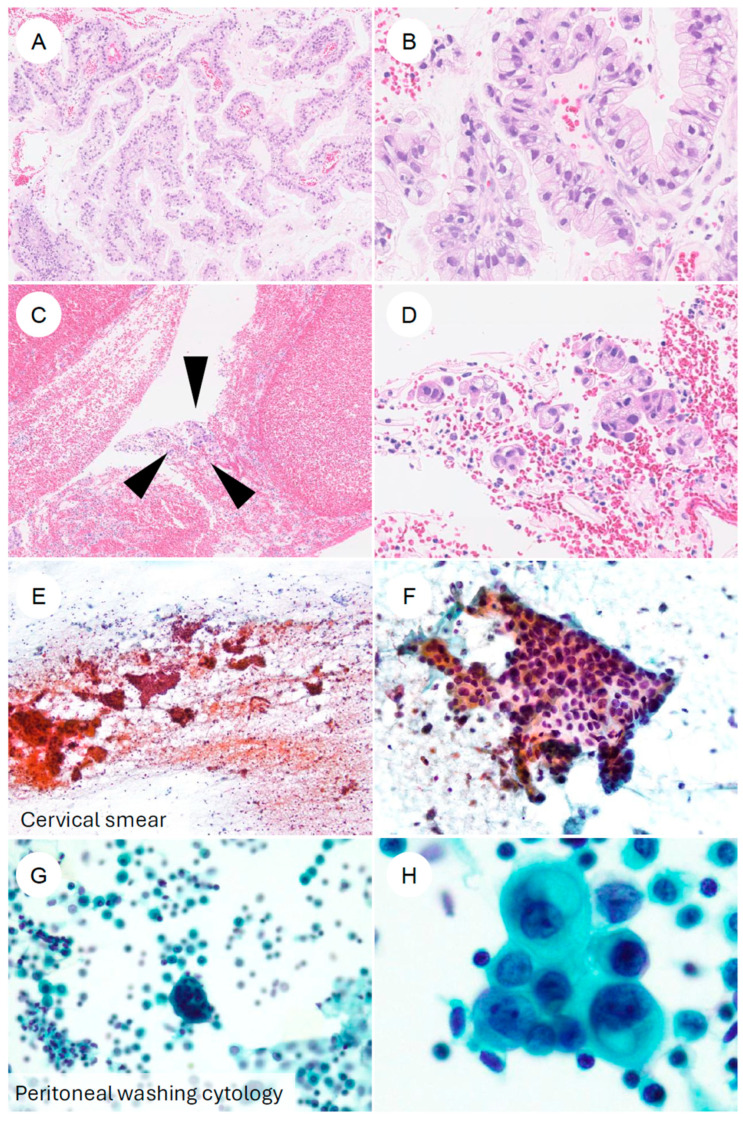
Cytologic and biopsy appearances of gastric-type endocervical adenocarcinoma. (**A**,**B**) Cervical punch biopsy of gastric-type adenocarcinoma. At low magnification, numerous atypical epithelial cells form papillary structures. At higher magnification, the tumor is composed of cells with enlarged, hyperchromatic nuclei and clear to foamy cytoplasm, with sharply defined cell borders, arranged in complex papillary proliferations. (**C**,**D**) Another cervical biopsy from gastric-type adenocarcinoma. In contrast to (**A**,**B**), only a scant number of tumor cells (black arrowheads) are present in a blood-rich background. At higher power, small clusters of tumor cells with eccentrically located, irregular nuclei and foamy cytoplasm can be identified. (**E**,**F**) Cervical smear cytology specimen. Against a hemorrhagic, necrotic background, irregular cell clusters are observed. At higher magnification, flat “honeycomb-like” sheets composed of tumor cells with nuclear enlargement, hyperchromasia, irregular nuclear contours, and loss of nuclear polarity are evident; some neoplastic cells contain characteristic golden-yellow intracytoplasmic mucin. (**G**,**H**) Ascitic fluid cytology from a patient with gastric-type adenocarcinoma. In a background containing inflammatory cells and reactive mesothelial cells, small clusters of atypical epithelial cells are seen. At higher magnification, adenocarcinoma cells with eccentrically placed, enlarged, irregular nuclei and mucin-containing cytoplasm are identified.

Cervical cytology (Pap smear) has low sensitivity for detecting GAS, and is often misinterpreted as “atypical glandular cells (AGC)” or similar categories [20,64,65,66,67,68]. Nevertheless, several cytomorphologic features distinct from those of UEA have been reported (Figure 5) [20]. Compared with the three-dimensional cellular clusters commonly seen in UEA, GAS more frequently forms flat, monolayered honeycomb sheets [20,65,66,68,69]. Single, dispersed tumor cells and cells resembling signet-ring cells may also be observed [7,20,67,69]. The cytoplasm is typically abundant, vacuolated, or foamy, with well-defined cell borders [20,66,67,68,69]. Reflecting the gastric-type mucin, the presence of golden-yellow to brown intracytoplasmic mucin (golden-yellow mucin) can serve as a diagnostic clue [20,65,68,70]. The nuclei tend to be basally located, with vesicular chromatin and prominent nucleoli [20,66,67,69]. PWC is considered a potential adverse prognostic factor in cervical cancer [18], and in GAS, PWC positivity has also been associated with poor outcomes [18]. In PWC specimens, GAS cells frequently form spheroid clusters and typically lack the yellow mucin seen in cervical smears [18]. In addition, although rare, cases of locally invasive GAS detected on urine cytology have been reported [71].

### 3.2. Immunohistochemical Features (Figure 6)

Integration of morphologic features with the immunohistochemical profile helps diagnose GAS [4,13,37].

**Figure 6:**
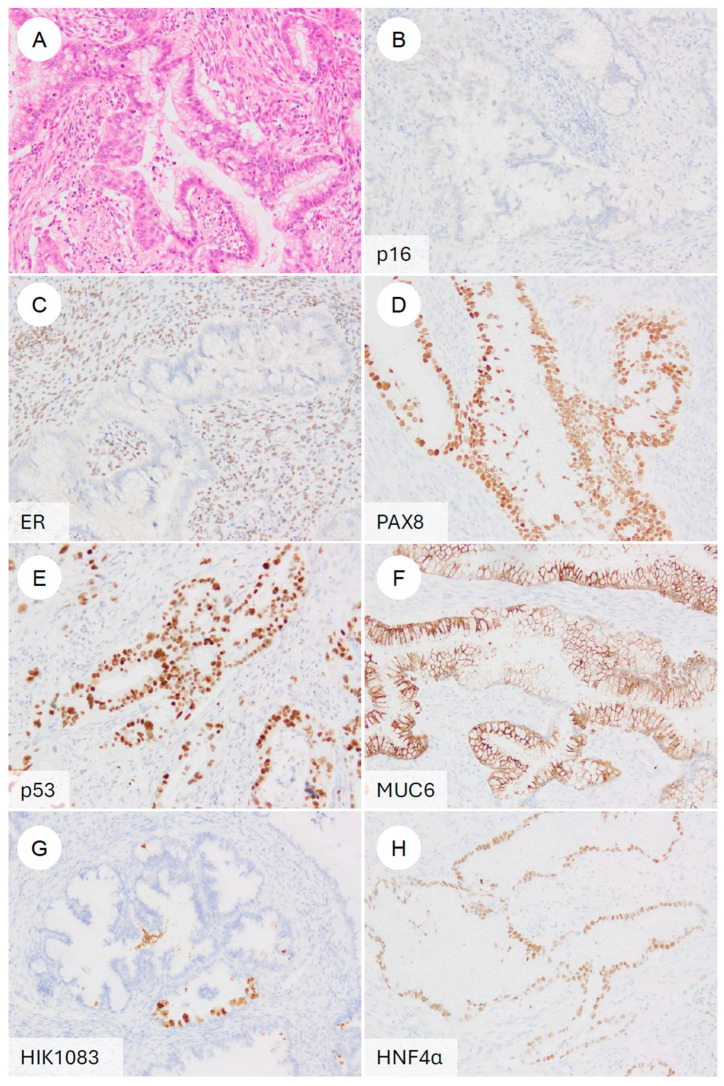
Typical immunophenotype of gastric-type endocervical adenocarcinoma. (**A**) H&E stain. A representative gastric-type adenocarcinoma showing classic morphologic features. On immunohistochemistry, the tumor is (**B**) p16-negative (non-block-type), (**C**) ER-negative, (**D**) PAX8-positive, (**E**) p53 diffusely positive (mutant pattern), (**F**) MUC6-positive, (**G**) HIK1083-positive, and (**H**) HNF4α-positive.

#### 3.2.1. Diagnostic Markers

First, as a high-risk HPV-surrogate marker, GAS typically shows p16 negativity or a mosaic/non-block-type heterogeneous staining pattern [7,60,72]. This contrasts with the diffuse, strong block-type positivity characteristic of UEA [2,72]. In 81.0% of GAS cases and in 89.3% of tumors in another series, p16 is negative or only patchily positive [37]. In contrast, some studies have reported diffuse block-type p16 positivity in 23.3% of GAS [73]. However, these exceptional cases have frequently been found to harbor p53 abnormalities [73]. Even in such GAS cases showing block-type p16 positivity, high-risk HPV DNA is not detected by HPV-ISH [5,72,74]. More recently, it has been reported that even in p16-positive GAS, Rb expression is preserved, and that the combined assessment of Rb and p16 may help distinguish these tumors from HPV-positive adenocarcinomas, which consistently demonstrate the partial loss of Rb [75].

While p16 immunohistochemistry serves as a useful surrogate, it is not definitive for determining HPV status. Strict confirmation of HPV independence requires molecular testing. Although HPV in situ hybridization (ISH) is frequently used in routine pathology practice, it has lower sensitivity compared to polymerase chain reaction (PCR)-based methods, which may lead to false-negative results [74]. Therefore, highly sensitive PCR-based HPV genotyping is considered the most reliable method for ruling out high-risk HPV infection. Furthermore, recent large-scale genomic studies have leveraged next-generation sequencing (NGS) data (e.g., RNA-seq) to bioinformatically confirm the absence of viral reads, thereby providing robust evidence of HPV independence in GAS [76].

Estrogen receptor (ER) and progesterone receptor (PR) are typically negative in GAS [7,14], a feature that helps distinguish it from isthmic endometrioid carcinoma with mucinous metaplasia and from serous carcinoma. In contrast to the relative rarity of p53 abnormalities in UEA [37,38], abnormal (mutant-type) p53 staining patterns in GAS, showing either diffuse strong overexpression or complete absence, have been reported in approximately 50% of cases [37,38].

To confirm gastric differentiation, the expression of gastric (pyloric gland)-type mucins or cell adhesion molecules is commonly evaluated [7,14,15,63,73,77]. MUC6, a marker of pyloric gland mucin [14,63,73], is frequently positive in GAS (up to ~89%). Although its sensitivity is high, MUC6 can also be expressed in normal endocervical glands and in UEA, resulting in limited specificity [5,73]. HIK1083 was initially reported as a highly sensitive marker for gastric-type mucin [78,79]; however, accumulating data indicate that, although its sensitivity is more modest (~67.7%), its specificity is relatively high [73]. HIK1083 has been used in the earliest studies in association with MDA, a precursor lesion within the GAS spectrum [14,63,79]. In addition, TFF2 is a gastric marker comparable to MUC6 and HIK1083, and double positivity for TFF2 and HIK1083 confers high specificity for GAS [80].

More recently, claudin 18.2 (CLDN18) has attracted attention as a highly sensitive and specific marker for gastric-type differentiation [73,77,81], showing diffuse positivity in tumor cells of GAS. CLDN18.2 is a tight junction protein enriched in gastric epithelium [15,73,81]. In GAS, the reported positivity rate is 72–95%, whereas UEA and benign cervical lesions are negative, making CLDN18.2 highly specific for GAS [15,73,77,81]. Overexpression of claudin 18.2 in GAS has been suggested to be regulated by genomic hypomethylation of its promoter CpG islands [81]. In gastric cancer, CLDN18.2 is the target of a monoclonal antibody (zolbetuximab) that has entered standard clinical practice [15,81], and given its high expression in GAS, CLDN18.2 is also being explored as a promising novel therapeutic target in this tumor type [15,77,81].

In addition, hepatocyte nuclear factor 1β (HNF1β) was expressed in 91% of GAS cases (21/23) and in 100% of gAIS cases (3/3) [82]. Moreover, trichorhinophalangeal syndrome type 1 (TRPS1), a recently described breast cancer marker, has also been reported to be expressed in gynecologic malignancies, including GAS [83], and may aid in identifying the primary site when GAS presents as a metastasis [83].

#### 3.2.2. Potential Therapeutic Targets and Immune Markers

Other potential therapeutic targets include HER2. HER2 overexpression (score 3+) has been identified in approximately 3.7–25% of GAS cases [15,77], suggesting that GAS may be amenable to anti-HER2-directed therapy [15]. HER3 is also overexpressed in GAS tissue [84]; in one study, all 10 evaluated cases showed expression of at least 2+ intensity [84]. These findings suggest that ADC therapies targeting HER3 may represent a promising treatment option [84].

The expression of immune checkpoint molecules has also been investigated in GAS [15,81,85,86]. Chen et al. reported no statistically significant differences in PD-L1 expression among histologic subtypes of cervical adenocarcinoma, including GAS, when evaluated using either the combined positive score (CPS) or the tumor proportion score (TPS) [86]. However, when TPS was used, PD-L1-positive GAS showed significantly worse PFS and OS than PD-L1-negative GAS [86]. Lin et al. found PD-L1 positivity in 37% of 46 GAS cases using a CPS cutoff of 1% [81]. In another cohort, PD-L1-positive tumors (CPS-positive: 50/107 cases) had significantly shorter PFS and OS than PD-L1-negative tumors, as assessed by TPS [15,86]. Sun et al. analyzed the expression of T-cell immunoglobulin and mucin-domain containing-3 (TIM-3) and B7 homolog 3 (B7-H3) in 58 GAS cases, and, using a TPS cutoff of 1%, reported expression of B7-H3 and TIM-3 in 48.3% and 17.2% of tumors, respectively [85]. The expression patterns of the three checkpoints (B7-H3, TIM-3, and PD-L1) did not completely overlap, and patients whose tumors were positive for B7-H3 or TIM-3 by TPS had significantly shorter RFS and OS [85].

### 3.3. Differential Diagnosis

Accurately distinguishing GAS from its mimics is critical for appropriate clinical management. The primary differential diagnosis includes metastatic adenocarcinoma from the gastrointestinal (GI) and pancreatobiliary tracts, which can morphologically mimic GAS. To rule out metastasis, immunohistochemistry is essential; GAS is typically positive for PAX8 (68–88%), whereas GI adenocarcinomas are consistently PAX8-negative [38]. Clinical correlation with imaging and endoscopic findings is also required to exclude a non-cervical primary.

Other differentials include usual-type endocervical adenocarcinoma (UEA), clear cell carcinoma (CCC), and mesonephric lesions. Unlike GAS, UEA typically shows diffuse p16 positivity and is HPV-positive. CCC can be distinguished by Napsin A expression, which is frequently negative in GAS but positive in CCC [82]. Mesonephric lesions typically retain PAX2 expression, whereas PAX2 is frequently lost in GAS [87].

## 4. Molecular Landscape of GAS

GAS is a rare adenocarcinoma that arises independently of HPV infection, and its low incidence has historically limited the availability of comprehensive genomic analyses. Most earlier reports did not focus on GAS alone but instead included it as part of comparative studies with HPVA or were restricted mainly to IHC or single-gene analyses. More recently, however, the introduction of next-generation sequencing (NGS)-based transcriptomic profiling and multigene panel testing has begun to clarify the molecular pathogenesis of GAS in greater detail.

In an earlier NGS-based study, Garg et al. analyzed fourteen cases of GAS and identified a mean of seven mutations per tumor. The most frequently mutated gene was *TP53*, followed by *MSH6*, *CDKN2A/B*, *POLE*, *SLX4*, *ARID1A*, *STK11*, *BRCA2*, and *MSH2*. Overall, mutations were detected in genes involved in DNA damage and repair, cell cycle regulation, the Fanconi anemia pathway, and the PI3K–AKT signaling pathway [38]. Park et al. subsequently analyzed 21 GAS cases by NGS and reported a mean of 2.6 mutations per lesion (range, 0–9). The most frequently altered gene was *TP53* (11/21, 52.4%), followed by *STK11*, *HLA-B*, and *PTPRS* (4/21, 19.0%), *FGFR4* (3/21, 14.3%), and *GNAS*, *BRCA2*, *ELF3*, *ERBB3*, *KMT2D*, and *SLX4* (2/21, 9.5%). Additional genes, including *CDH1, EPCAM, KRAS*, *MLH1*, *RNF43*, *SNAI1*, *TWIST1*, *ZEB1*, and *ZEB2*, were each mutated in 1 of 21 cases (4.8%). Many of these alterations involved genes related to signal transduction, DNA damage repair, and EMT [37]. Lu et al. performed NGS in 15 GAS cases and again found *TP53* to be the most frequently mutated gene (8/15, 53.3%), followed by *STK11, CDKN2A*, and *ARID1A* [36]. *STK11* mutations were significantly more frequent in well-differentiated GAS (33.3% vs. 0.0%, *p* = 0.026) and in patients with extensive LVSI (33.3% vs. 0.0%, *p* = 0.044). Furthermore, *STK11* mutation was associated with poor outcomes in GAS [36]. Potentially targetable alterations were identified in 53.3% of GAS patients, and, according to OncoKB annotations, *ERBB2* amplification (13.3%) had the highest level of evidence [36]. Jung et al. compared genomic abnormalities between UEA and GAS, reporting that, in GAS, mutations were found in *KRAS*, *TP53*, *NF1*, *CDKN2A*, *STK11*, and *ARID1A*, with *MDM2* amplification detected in one case. By contrast, in UEA, mutations were observed in *HRAS*, *PIK3CA*, and *BRCA2* [22].

In recent years, Yang et al. have reported consistently high claudin 18.2 expression in GAS [81], demonstrating that this molecule is not only a useful diagnostic marker, but also a promising therapeutic target [15,81]. Vasseur et al., using RNA-sequencing-based analysis, showed that protumorigenic pathways such as WNT, PI3K/AKT, and TGF-β are activated in GAS and reported the additional upregulation of transcription factors involved in immunosuppression and EMT [88]. Ehmann et al., through comprehensive genomic analyses across several cohorts including a total of 70 GAS cases, confirmed frequent alterations in genes such as *TP53* and *CDKN2A* [16], and further identified mutations in DNA repair-related genes, including *BRCA2* and *PALB2*, in a subset of tumors [16], suggesting that GAS may arise through diverse molecular pathways [16].

Higuchi et al. performed a multi-omics analysis on a relatively large series of 99 GAS cases and comprehensively delineated the molecular differences between GAS and UEA [76]. Their analysis showed coordinated alterations at the genomic, transcriptomic, and metabolic levels, and demonstrated that GAS is characterized by distinctive metabolic reprogramming and dysregulation of cell cycle control [76].

Collectively, these studies indicate that GAS is defined by multilayered molecular abnormalities involving cell cycle regulation, DNA repair, signal transduction, epigenetic regulation, and metabolism. Recent comprehensive analyses have further clarified that these alterations are closely linked to the aggressive clinical behavior and treatment resistance of GAS. Below, we focus on the most frequently altered genes and summarize their roles.

*TP53* mutations are among the most frequent molecular abnormalities identified in GAS [22,36,37,38,76]. In the series reported by Ehmann and Jung and their colleagues, TP53 mutations were detected in approximately 50–65% of cases [16,22,36,37,38,76]. A similar frequency was confirmed in the cohort reported by Higuchi et al., which included cases from C-CAT and GENIE [76]. The vast majority of these alterations are missense or truncating mutations [36,37,38], resulting in the loss of p53 function and consequent impairment of cell-cycle arrest, apoptosis, and DNA repair pathways [76]. By integrating RNA expression and metabolomic analyses, Higuchi et al. demonstrated that *TP53* mutations are closely linked to metabolic pathway reprogramming in GAS [76]. In particular, marked downregulation of *ISYNA1*, a gene involved in inositol metabolism, was characteristic of GAS and correlated with *TP53* mutations [76]. Mechanistically, ISYNA1 encodes the rate-limiting enzyme for myo-inositol biosynthesis. Its downregulation leads to the intracellular depletion of myo-inositol, which may subsequently alter membrane composition and signaling stability, thereby contributing to the aggressive phenotype and chemoresistance of GAS, although the precise downstream effects remain under investigation. Furthermore, cases harboring *TP53* mutations showed abnormalities in tricarboxylic acid (TCA) cycle-related metabolites [76], indicating that *TP53* alterations also shape the metabolic phenotype of GAS. Immunohistochemically, *TP53* mutations are most often reflected by aberrant p53 staining patterns, and Jung et al. have reported the diagnostic utility of this finding [22].

*CDKN2A* mutations are also frequent in GAS, being detected in approximately 30–40% of cases in the previous studies [16,36,37,76]. These alterations disrupt G1/S cell-cycle control via the p16 pathway and promote sustained proliferative signaling in tumor cells [76]. *KRAS* mutations are detected in roughly 15–20% of tumors, predominantly as activating missense substitutions such as G12D and G13D [16,36,37,38,76,81]. These mutations contribute to enhanced tumor growth and invasiveness by aberrantly activating the PI3K/AKT and MAPK pathways [36,37]. *STK11* mutations have also been reported in a subset of cases and are thought to be associated with disturbances in cellular metabolism and polarity control [36,37,38]. In addition, germline *STK11* mutations underlying Peutz–Jeghers syndrome have been reported in approximately 10% of patients with GAS [25]. Mechanistically, STK11 (also known as LKB1) functions as a negative regulator of the mTOR signaling pathway. Consequently, loss-of-function mutations in STK11 lead to the hyperactivation of mTOR signaling, which drives aberrant cell growth and metabolic reprogramming. This pathway deregulation is considered a central driver in the pathogenesis of Peutz–Jeghers syndrome-associated GAS.

Ehmann et al. have also reported mutations in genes such as *ARID1A*, *KMT2D*, and *SMAD4*, which are involved in chromatin remodeling and transcriptome regulation [16,36,37,81], suggesting disruption of epigenetic control in GAS [16]. In addition, some cases harbor mutations in homologous recombination repair deficiency (HRD)-related genes, including *BRCA2*, *ATM*, and *PALB2* [16,36,37,38,81], suggesting that HRD may contribute to tumorigenesis in a subset of GAS [16,36,37,81]. These findings raise the possibility that therapeutic interventions, such as PARP inhibition, may be effective in selected cases [81,89].

RNA-seq-based analyses have demonstrated that GAS exhibits a distinct transcriptional profile compared with HPVA [76,88]. Higuchi et al. showed that GAS clusters within a p53-suppressed transcriptional subtype, characterized by metabolic reprogramming centered on ISYNA1 [76]. Vasseur et al. reported consistent activation of WNT/β-catenin, TGF-β, and EMT-related genes in GAS, implicating these pathways in metastasis and immune evasion [88]. Ehmann et al. further described an immunosuppressive tumor microenvironment in GAS, reflected by the downregulation of immune-response-related genes [16].

Taken together, the oncogenesis of GAS is driven by multilayered molecular abnormalities. Alterations in cell cycle regulators, primarily *TP53* and *CDKN2A*, lead to unrestricted proliferation and genomic instability [37,38,76]. The *PI3K*/*AKT*/*mTOR* pathway is frequently activated, often via *KRAS* mutations, supporting cell survival and invasion [16,37,38]. Additionally, transcriptomic evidence indicates that WNT/β-catenin and TGF-β signaling pathways are activated, contributing to EMT and metastasis [88], while mutations in chromatin remodelers like *ARID1A* and *KMT2D* imply epigenetic dysregulation [16,38]. These diverse alterations collectively shape the aggressive and immunosuppressive phenotype of GAS [16,88].

In summary, tumor progression in GAS is tightly linked to abnormalities in cell cycle regulation, metabolism, signal transduction, epigenetic control, DNA repair, and immune pathways, each contributing to the full spectrum of events from tumor initiation and progression to therapeutic response. A deeper understanding of these molecular mechanisms will be essential for developing prognostic models and designing personalized therapeutic strategies.

## 5. Therapeutic Implications

GAS is characterized by resistance to conventional therapies and an aggressive clinical course; thus, an optimal, evidence-based treatment strategy has yet to be established [13,34,35,41,90]. At present, management relies on treatment guidelines developed for cervical SCC and UEA [91]. However, there is an urgent need to define therapeutic approaches tailored to the biology of GAS [91,92].

### 5.1. Current Standard of Care

For early-stage (Stage I) GAS, surgery remains the cornerstone of treatment [91,93]. In accordance with standard cervical cancer guidelines, radical hysterectomy with pelvic lymph node dissection is generally performed [91,93,94]. For Stage IA1 disease, a modified radical hysterectomy may be selected. Because GAS shows an unusual pattern of spread with a high propensity for ovarian metastasis and peritoneal dissemination [4,13,16,91], the removal of both uterine adnexa (ovaries and fallopian tubes), omentectomy, and resection of intraperitoneal metastatic lesions are sometimes recommended [91,93]. Given this strong propensity for ovarian metastasis, ovarian-preserving surgery may be contraindicated in the management of GAS [93]. Even in early-stage disease, fertility-sparing surgery (trachelectomy) is generally discouraged for invasive GAS due to its highly infiltrative nature [91]. However, it may be carefully considered in exceptional circumstances, such as in select cases of gAIS, provided that rigorous preoperative evaluation and counseling are performed. Given the marked treatment resistance of GAS [34,90], complete surgical resection is regarded as a critically important component of the therapeutic strategy [35,91]. By contrast, there is almost no evidence to guide the optimal extent of resection in cases where gAIS is suspected.

In patients with postoperative high-risk factors, such as lymph node metastasis or parametrial invasion, concurrent chemoradiotherapy (CCRT) or radiotherapy is recommended [90]. Cisplatin-based CCRT is the standard adjuvant or definitive treatment [90,91]. In Japan, to avoid gastrointestinal and genitourinary toxicity associated with CCRT, chemotherapy alone is sometimes selected as a postoperative adjuvant therapy [90]. GAS has been repeatedly shown to exhibit marked resistance to standard treatment when compared with HPVA [4,13,34,36,90]. A characteristic feature of GAS is its chemoresistance [34,37], and postoperative adjuvant chemotherapy is often associated with poor outcomes in patients with GAS [90]. In patients with recurrent or metastatic GAS treated with chemotherapy, response rates have not been superior to those observed in UEA [13]. Based on genomic analyses, frequent alterations in EMT-related genes in GAS have been implicated as potential contributors to its aggressive clinical behavior and chemoresistance [22,34,37]. Furthermore, even when combined with anti-angiogenic agents such as bevacizumab, chemotherapy has not resulted in significant improvements in PFS or OS in patients with GAS, suggesting that anti-angiogenic therapy may not be a favorable treatment option in this setting [95].

GAS has also been reported to exhibit resistance to radiotherapy [13,34,41,90]. Nishio et al. demonstrated that among recurrent patients (12 cases of GAS, 11 cases of UEA), the response rate to radiotherapy was 50.0% (6 out of 12 cases) for GAS, whereas it was 81.8% for UEA, indicating that GAS was significantly resistant to radiotherapy [13]. Kuruma et al. (2022) reported that in patients with adenocarcinoma who underwent definitive RT, the 3-year PFS rate for GAS was 44.4%, which was significantly lower than the 3-year PFS rate for adenocarcinoma of other histological types (66.7%) [41,91]. In a cohort of postoperative high-risk patients, outcomes tended to be better with radiotherapy alone than with chemotherapy or CCRT [90]. This finding may reflect the intrinsic resistance of GAS to the chemotherapy component of CCRT [34,90]. In summary, while no GAS-specific standard exists, platinum-based chemoradiotherapy or chemotherapy is generally preferred over radiotherapy alone for high-risk cases, given the tumor’s potential radioresistance.

### 5.2. Emerging Therapeutic Approaches

Because GAS is refractory to the existing treatments [13,34,36,41,90], the development of novel therapeutic approaches incorporating ADCs and immunotherapy is considered essential [16,36]. Tisotumab vedotin (TV) is an antibody–drug conjugate (ADC) targeting tissue factor (TF). Based on results from the single-arm phase 2 innovaTV 204 trial in patients with previously treated metastatic or recurrent cervical cancer [96], it received FDA-accelerated approval in 2021, becoming the first ADC available for gynecologic cancer patients. Subsequently, the multinational phase 3 innovaTV 301 trial demonstrated the efficacy of TV over the treatment of physician’s choice [97] and received full FDA approval in 2024. In this trial, 33.6% of patients enrolled had adenocarcinoma. Although subgroup analysis did not suggest differences in efficacy based on histology, detailed pathological classification among adenocarcinoma patients was not reported. To our knowledge, only two cases exist regarding TV administration for GAS [98]. One case showed complete response on CT after 4 cycles and maintained response for at least 8 months [98], and in another case, the best overall response was progressive disease [99]. Data on GAS patients receiving TV is scarce, and its clinical utility remains poorly understood; the accumulation of real-world data is anticipated in the future. A subset of GAS, similar to breast and gastric carcinomas, harbors abnormalities in the HER2/HER3 signaling axis [16,37,81]. *HER2* (*ERBB2*) amplification or overexpression (IHC 3+) has been reported in 5–25% of GAS cases [21,77,81]; in one series, the amplification rate was 13.3% (2/15 cases) [36], and in another, it was 18.2% (2/11 cases), while HER2 overexpression (2+ or 3+) has been documented in approximately 20–25% of tumors (IHC 3+) [21,77]. Cases have been reported in which GAS patients with HER2 alterations received trastuzumab in combination with chemotherapy, followed by maintenance trastuzumab, achieving favorable clinical outcomes [16,81]. In the DESTINY-PanTumor02 trial of trastuzumab deruxtecan (T-DXd) in HER2-expressing solid tumors [100], efficacy was demonstrated in HER2-positive cervical cancer, with response rates of 75% in HER2 3+ and 40% in HER2 2+ tumors [100]. Two cases of GAS were included, but the detailed data are not available [99]. Given its activity even in tumors with low HER2 expression [101], T-DXd may be a promising therapeutic option for GAS. As T-DXd has shown efficacy in tumors with low-level HER2 expression (HER2 1+) [101], it may also offer a new treatment paradigm for GAS patients with low HER2 expression (1+), which is observed in 35.2% of cases [15]. A case series exists describing three patients who received T-DXd as second-line therapy for GAS. While no new safety concerns were identified, the therapeutic effect was reported to be limited [99]. HER3 (ERBB3) is likewise frequently overexpressed in GAS tissue [37,84], and the development of HER3-targeted ADCs and related agents is keenly anticipated [84]. Kojima et al. have established two GAS patient-derived xenograft (PDX) models, demonstrating preservation of the original morphologic and immunophenotypic features and suggesting that HER3 may represent a promising therapeutic target in GAS [84].

Immune checkpoint inhibitors (ICIs) are also a promising therapeutic option for GAS [81,85]. As described in the IHC section, PD-L1 expression is frequently observed in GAS [15,86]. This PD-L1 expression profile suggests that the PD-1/PD-L1 axis may be an attractive therapeutic target [81,86]. In the KEYNOTE-A18 trial, the addition of pembrolizumab to standard CCRT for locally advanced cervical cancer showed statistically significant and clinically meaningful survival benefits [102], and the pembrolizumab group included 18% non-squamous cell carcinoma, but the details regarding the proportion of GAS are unknown. In recurrent or metastatic setting, ICIs with platinum-based chemotherapy with or without bevacizumab is now regarded as a standard first-line systemic therapy, especially when PD-L1-positive, based on a phase 3 KEYNOTE-826 trial [103]. This approach may also apply to patients with GAS, though specific data on GAS does not exist. In second-line or later settings without a previous history of administration of ICIs, an anti-PD-1 antibody, cemiplimab, is one of the standard treatments based on the results from the EMPOWER-Cervical 1/GOG-3016/ENGOT-CX9 study [104]. In this trial, cemiplimab showed a statistically significant OS benefit regardless of histologic type (adenocarcinoma or adenosquamous carcinoma: 22.2%). Other relevant ICIs include agents targeting B7-H3 and TIM-3 [85]. Because their expression partially overlaps with that of PD-L1, therapeutic strategies targeting B7-H3 or TIM-3 are also considered promising [85].

Given the biological similarities between GAS and gastric carcinoma, additional therapeutic targets have attracted attention [81]. Claudin 18.2 is positive at a very high frequency in GAS (71.7–95.2%) [81], and therefore anti-claudin 18.2 monoclonal antibodies under development for gastric cancer such as zolbetuximab [105] have been proposed as potential therapeutic agents for GAS. Zolbetuximab, a monoclonal antibody targeting claudin 18.2, has shown favorable results in clinical trials for gastric and gastroesophageal junction adenocarcinomas [105,106]. Anti-claudin 18.2 therapy has thus been proposed as an attractive future research direction with the potential to improve outcomes in patients with GAS [15,73]. However, no clinical trials have yet evaluated their efficacy specifically in cervical GAS, and dedicated trials are eagerly awaited.

In addition, although they represent only a subset of GAS, cases harboring distinctive genetic alterations may offer additional therapeutic targets. For example, somatic mutations in HRD-related genes have been identified in a proportion of GAS [37]. There is a case report of an advanced GAS patient with a *BRCA* mutation in whom maintenance therapy with olaparib (a PARP inhibitor) successfully suppressed tumor progression [89]. Moreover, a phase II trial is ongoing in patients with advanced GAS harboring *STK11* mutations, evaluating a combination of a PI3K–mTOR cell-cycle inhibitor (WX390) and a PD-L1 inhibitor (toripalimab) [91]. Although *KRAS* mutations are present only in a subset of GAS [36,37], *KRAS* p.G12C-targeted inhibitors such as sotorasib may provide therapeutic opportunities for this small molecularly defined subgroup [16].

In summary, GAS has markedly poorer outcomes than HPVA and exhibits high-level resistance to conventional therapies. Consequently, an NGS-based definition of its molecular profile and the implementation of precision medicine strategies targeting molecules such as HER2/HER3, PD-L1, and claudin 18.2 are likely to be indispensable for improving outcomes in this challenging disease.

## 6. Future Perspectives and Unmet Needs

To improve future care for patients with GAS, sustained research efforts to enhance diagnostic accuracy, elucidate disease biology, and develop novel therapeutic strategies are indispensable.

First, clarification of the molecular pathogenesis and the development of new biomarkers are urgently needed [92]. To overcome the invasive nature of GAS and identify actionable therapeutic targets, it is essential to develop a detailed understanding of its specific molecular mechanisms [36,37,76,81,88]. In particular, the etiologic pathways of tumor development must be defined by identifying the key molecular events underlying neoplastic transformation in LEGH and gAIS to establish biomarkers for early detection [5,107]. Furthermore, comprehensive multi-omics analyses are required to elucidate the functional consequences of characteristic genomic alterations in GAS and to interrogate the contributions of signaling pathways, such as EMT pathways and p53 signaling, to its invasiveness and chemoresistance [34,36,37,76,81,88].

Although such work has scarcely been undertaken in GAS to date, investigation of the intratumoral microbiome also represents an important future direction [108]. It will be necessary to characterize the composition and role of the intratumoral microbiota in HPVIs and to clarify how these microbial communities influence tumor initiation and progression. Notably, recent data indicate that HPVIs harbor an intratumoral bacterial profile distinct from that of HPVAs, and that a prognostic model constructed from intratumoral microbiome signatures improved survival prediction compared with models based solely on conventional clinicopathologic factors [108]. In addition, as exemplified by the work of Higuchi et al., further efforts are needed to define the metabolic profile of GAS and to clarify how GAS-specific metabolic abnormalities, such as alterations in glutamine, proline, and inositol metabolism, contribute to tumor growth, thereby exploring their potential as therapeutic targets [76].

Next, improvements in diagnostic accuracy and the establishment of clear treatment indication criteria are needed. GAS is difficult to detect at an early stage [45], and highly differentiated tumors (MDA in particular) are prone to being misdiagnosed as benign lesions [5,7,109,110,111], making the development of reliable diagnostic methods critically important. To establish reproducible diagnostic approaches, algorithms must be developed that can accurately distinguish GAS/MDA on cytology and limited biopsy specimens [65,66,69,112], and differentiate them from benign lesions such as LEGH [50,73,77]. In addition, the actual clinical behavior (rates of metastasis and recurrence) of morphologically deceptively benign MDA should be investigated in larger cohorts [52,113], in order to refine indications for the extent and invasiveness of treatment, including the need for lymph node dissection and adnexal removal [52]. Furthermore, improving the performance of imaging-based diagnosis is an important task; additional work is required to correlate MRI characteristics with pathological findings to mitigate the current tendency to underestimate tumor extent and patterns of spread, including parametrial invasion, ovarian metastasis, and peritoneal dissemination [33,42,45,46,114]. Moreover, the development of radiomic signatures and radiogenomic studies investigating the correlation between imaging parameters (e.g., ADC values) and specific molecular alterations (e.g., *TP53* or *KRAS* mutations) represent anticipated areas of research to improve preoperative diagnostic accuracy.

Clinical validation of novel therapeutic strategies is also indispensable. To address the treatment resistance of GAS, there is a pressing need for prospective clinical trials evaluating the efficacy of molecular targeted agents and immune checkpoint inhibitors [15,85,86]. However, given the rarity of this tumor, single-institution studies are insufficient; thus, international collaborative trials are a prerequisite for validating these targeted therapies and establishing high-level evidence. Priority areas include clinical validation of claudin 18.2-targeted therapy [15,73], prospective evaluation of HER2/HER3-targeted approaches [84], the adaptation of treatment regimens used for molecularly similar gastric and pancreatobiliary carcinomas [16,81], and the further exploration of site-agnostic use of PARP inhibitors (e.g., olaparib) in GAS patients harboring mutations in DNA repair-related genes [89]. In addition, it will be essential to assess the efficacy of ICI monotherapy or combination immunotherapy in GAS, which frequently expresses immune checkpoint molecules such as PD-L1, B7-H3, and TIM-3 [85].

Finally, given the rarity of GAS, strengthening research infrastructure is crucial. Large-scale registry development is required to accumulate clinical outcome data and to build more robust management guidelines for this rare entity, through international cancer registries that systematically collect clinical data, pathological features, and genetic alterations [16]. The use of PDX models should also be actively promoted. Because GAS PDX models preserve intratumoral heterogeneity while allowing pharmacologic responses to be evaluated [84], they should be leveraged as key platforms for preclinical testing of novel therapeutic agents.

## 7. Conclusions

GAS is a biologically and clinically distinct, HPV-independent cervical adenocarcinoma characterized by aggressive behavior, a unique pattern of dissemination, and pronounced resistance to standard chemoradiotherapy. Integrative genomic and multi-omics studies have delineated a complex network of abnormalities in cell-cycle regulation, signal transduction, metabolism, epigenetic control, DNA repair, and immune pathways that collectively drive its invasive growth and treatment resistance, while also revealing actionable vulnerabilities such as HER2/HER3 signaling, claudin 18.2 overexpression, immune checkpoint activation, and homologous recombination deficiency. However, early detection remains difficult, diagnostic reproducibility is suboptimal, particularly for highly well-differentiated lesions, and robust evidence to guide GAS-specific staging, surgical extent, and systemic therapy is lacking. Future progress will depend on the development of reliable diagnostic algorithms and biomarkers, the prospective evaluation of targeted and immune-based therapies, and the establishment of international registries and preclinical models to support adequately powered, biology-driven clinical trials for this rare but increasingly important tumor type.

## Data Availability

No new data were generated or analyzed in this study. Data sharing is not applicable to this article.
